# Bacteriological and cytological findings during the late puerperal period after two different treatments of retained placenta followed by acute puerperal metritis

**DOI:** 10.1186/1751-0147-52-41

**Published:** 2010-06-15

**Authors:** Julia Jeremejeva, Toomas Orro, Merle Valdmann, Kalle Kask

**Affiliations:** 1 Department of Therapy, Institute of Veterinary Medicine and Animal Sciences, Estonian University of Life Sciences, Kreutzwaldi 1, Tartu, 51014, Estonia; 2 Department of Animal Health and Environment, Institute of Veterinary Medicine and Animal Sciences, Estonian University of Life Sciences, Kreutzwaldi 1, Tartu, 51014, Estonia

## Abstract

The aim of the study was to compare the effect of two acute puerperal metritis (APM) treatment protocols on uterine condition during the late puerperal period (5^th ^to 7^th ^week). Late gestation healthy cows (n = 21) were divided randomly in three equal groups. Parturitions were induced. Treatments of APM were started on the third day postpartum (PP). Group A was treated with an oxytocin analogue carbetocin for three days and intrauterine administration of cephapirin between days 15 and 17. Group B was given intramuscular injection of ceftiofur for five days followed by two injections of prostaglandin F_2α, _at an interval of 12 h, on the eighth day PP. Group C served as the control group with no treatment. Body temperature was recorded daily for 14 days PP. Uterine biopsies for bacteriology, and uterobrush samples for cytology, were taken once a week from the 5^th ^to 7^th ^week postpartum. No differences were found in body temperature on day 14 PP, presence of bacteriological infections and disappearance of uterine inflammatory signs diagnosed by cytological examination between experimental groups.

## Findings

Acute puerperal metritis (APM) is one of the most serious problems during the puerperal period for the dairy cow, which can cause high economic losses [1]. The main losses are related to low fertility, an increase in days open and involuntary culling from the herd [2]. Treatment of APM should reduce clinical signs (body temperature, eliminate signs of toxaemia and anorexia) and generally improve the well being of the animal. The most important purpose of the APM treatment is the elimination of uterine pathogens, which provides for quicker uterine involution and the recovery of normal uterine physiology.

Recent studies have compared different APM treatment methods [2,3]. The present study is the first report where intramuscular injections of ceftiofur in combination with a prostaglandin F_2α _(PGF_2α_) have been compared with administration of oxytocin during the earlier stage of inflammation in combination with intrauterine administration of cephapirin during the later stage of inflammation. The aim of the study was to compare the effect of the two APM treatment methods on bacteriological and cytological findings during the late PP period (5^th ^to 7^th ^week PP) in comparison with untreated control animals.

The study was conducted on a tie-stall commercial dairy farm with 450 cows. Twenty one multiparous late pregnant Estonian Holstein Friesian cows with an average milk yield of 10,250 kg energy-corrected milk during the previous lactation were used. In order to obtain retained foetal membranes (RFM) followed by APM, parturition was induced in all cows two weeks before term using two PGF_2α _injections (Dinolytic^®^, Pfizer Animal Health) according to the method described by Kask et al. [4]. Foetal membranes were defined as retained if they were not expelled within the first 24 hours after delivery [5].

No treatment of RFM was performed before the study manipulations. Diagnosis of APM was made on the third day postpartum (PP) using clinical examination. A diagnosis was confirmed if anorexia, enlarged and atonic uterus, and foul smelling watery red-brown vaginal discharge [6], were found. Considering the results from previous studies where in some cases no pyrexia was found in case of APM with severe bacterial infection [7,8], in our study increased body temperature (≥ 39.5°C), if detected, was used as an additional sign. Treatment was started immediately after diagnosis of APM (third day PP). The animals were randomly divided into three groups, seven in each group. Common treatment method used in the herd was introduced in the first group (A): 0.35 mg carbetocin (Hypophysin ^® ^LA, Veyx-Pharma GmbH, Schwarzenborn, Germany) intramuscular (i.m.) for three consecutive days, starting on the third day PP and single intrauterine administration of 500 mg cephapirin (Metricure^®^, Intervet International B. V, Boxmeer, the Netherlands) between days 15 and 17 PP. Animals from the second group (B) were treated by i.m. injections of 1 mg/kg ceftiofur (Excenell RTU^®^, Pfizer Manufacturing Belgium N.V., Puurs, Belgia) for five days followed by two i.m. injections of 25 mg dinoprost (Dinolytic^®^, Pfizer Manufacturing Belgium N.V., Puurs, Belgia) at an interval of 8 h on the eighth day PP. The third group (C) served as the control group without any treatment.

Body temperature (BT) was measured during the first two weeks PP.

Biopsies from uterine endometrium were collected from all animals once a week over a period from the fifth to the seventh week PP. To determine the species of the isolates the BBL Crystal™ (Becton, Dickinson and Company, Maryland, USA) miniaturized biochemical test systems (Gram positive, Enteric/nonfermenters and Anaerobe ID kits (BD BBL Crystal™ Identification Systems)) were used.

Uterine samples for cytological examination were collected weekly using a uterobrush (Uterobrush^®^, Medscand Medical AB, Malmö, Sweden), which was fastened onto a stainless steel device for use in cows. Slides for cytological examination were prepared by rolling the uterobrush onto a clean microscope glass slide. Slides were immediately fixed in a current of warm air using a blow drier. Slides were stained with May-Grünwald Giemsa stains. Cytological criteria for endometrial inflammation were set according to Kasimanickam et al. [9]. Briefly, >18% and >10% polymorphonuclear (PMN) cells at 20-33 and 34-48 days post partum (PP), respectively, were used to indicate inflammation. Non-parametric Kruskal-Wallis test followed by Tukey test with ranked sums for pair wise comparisons were used to explore differences in BT between the treatment groups at the day of diagnosing of APM (day 3 PP) and at day 14 PP. Group differences in bacteriological findings (bacteria present or not) and cytological investigation results (inflammation present or not) by weeks were tested using Fischer exact test. The statistical software Stata 9.2 (Stata Corp, Texas, USA) was used for all statistical analyses except WINKS SDA 6.0 (TexaSoft, Texas, USA) was used for Kruskal-Wallis test.

After induction of parturition all the animals calved between 268-276 days of gestation. All cows had retention of placenta and APM as a complication. One cow from group A was culled from the herd and excluded from the study during the third experimental week because of polyarthritis.

One animal from group A and three cows from group B did not show pyrexia during 14 days of experimental period. Body temperature on the day of diagnosing of APM (day 3 PP) in groups A, B and C was 39.5 ± 0.18°C, 38.6 ± 0.15°C and 39.2 ± 0.28°C respectively. Temperature in group A was significantly higher than in group B (P < 0.05). This difference was random, because dividing of animals to groups was random and treatment was started on the day 3 PP. At the end of BT measurement period (day 14 PP) BT in experimental groups did not differ (P = 0.056) and was 37.9 ± 0.07, 38.3 ± 0.06 and 38.4 ± 0.17 in A, B and C groups, respectively.

Intrauterine and parenteral antibiotics used in present study should reduce bacterial growth in the inflamed uterus. However many samples with heavy bacteriological growth were detected (Table 1). The most frequent isolates were *Bacteroides spp*. (52%) and *F. necrophorum *(30.5%). *Bacteroides spp. *were the major bacteria found in group B (80% of all the micro-organisms isolated in this group). The treatment did not have any effect on the elimination of these bacteria. Samitz et al. [10] reported poor effect of ceftiofur against *Bacteroides spp. *The main pathogen isolated in group A was *F. necrophorum *(75%). It is interesting that the growth rates of *F. necrophorum *in group A and *Bacteroides spp. *in group B were higher than in the non-treated group. A possible explanation could be the likelihood of more active regulation of such mechanisms as cytokines, the function of antibody production of leukocytes, and an endotoxin response in the non-treated animals [11].

**Table 1:** Number of bacteriologically positive samples and samples with cytologically demonstrable inflammation in experimental groups by weeks postpartum.

	Groups	Weeks postpartum		
		
		5	6	7
Number of bacteriologically positive samples	A	2/6	3/6	3/6
	B	4/7	5/7	1/6
	C	2/7	1/7	2/7

Number of samples with cytologically demonstrable inflammation	A	5/6	5/6	3/6
	B	7/7	3/7	3/7
	C	4/6	5/7	4/7

Results of the cytological examination are shown in Table 1. A total 59 cytological samples were collected. Cytologically diagnosed inflammation was found in 66.1% of samples. The majority of cows those were cytologically negative for subclinical endometritis in week 5 PP were also negative in weeks 6 and 7. The proportion of animals without cytological evidence of inflammation between groups by weeks was not significant.

A total of 56 cytological samples were taken simultaneously with uterine biopsies. One bacteriological sample in group B and one cytological sample in group C were missing due to problems during collection. Twenty samples out of 56 (35.7%) were with cytologically demonstrable inflammation and bacteriologically negative. Sixteen from fifty-six samples (28.6%) demonstrated cytological evidence of inflammation and were bacteriologically positive. In the same number of samples (16 from 56; 28.6%) we did not find inflammation using cytological criteria and did not isolate any bacteria. From 20 bacteriologically positive samples 4 (7.1% from total number of samples) did not demonstrate cytological evidence of inflammation. Previous studies showed that sensitivity of cytobrush cytology for diagnosing subclinical endometritis is high [9,12]. In the present study some samples with cytological evidence of inflammation also showed phagocytized bacteria, however uterine biopsies taken at the same time were found to be bacteriologically negative. Consequently bacteriological investigation of uterine biopsies in the present study could not detect all cases of bacteriological infection. In addition sterile uterine inflammation may also explain the discrepancies. We know, that the majority of uterine inflammations during puerperal period begin with bacterial contamination of the uterine lumen [6], but it is possible that after mobilisation of uterine defence mechanisms bacterial growth can be suppressed and inflammation can occur in the absence of active bacterial infection. Similar results have been described in beef cows after resumption of ovarian cyclicity [13]. It could be that detected uterine inflammations without isolation of bacteria might be caused by mycobacterial or fungal uterine contaminations that were not investigated in this study.

The findings of the present study indicate that the used treatments of APM had no effect on bacteriological and cytological findings during the late puerperal period. It is possible that no treatment may be the best option in the situation when APM occurs without severe depression, anorexia and high fever.

## Competing interests

The authors declare that they have no competing interests.

## Authors' contributions

JJ carried out the study, compiled the results and drafted the manuscript. TO participated in the designing the study and statistical analysis. MV performed cytological analysis and KK coordinated the study. All authors were significantly involved in designing the study, interpreting of data and composing the manuscript. All authors read and approved the final manuscript.
